# Identification of *Rickettsia felis* DNA in the blood of domestic cats and dogs in the USA

**DOI:** 10.1186/s13071-020-04464-w

**Published:** 2020-11-18

**Authors:** Md Monirul Hoque, Subarna Barua, Patrick John Kelly, Kelly Chenoweth, Bernhard Kaltenboeck, Chengming Wang

**Affiliations:** 1 Department of Pathobiology, Auburn University College of Veterinary Medicine, Auburn, AL 36832 USA; 2 Department of Clinical Sciences, Ross University School of Veterinary Medicine, Island Main Road, West Farm, Basseterre, Saint Kitts and Nevis

**Keywords:** *Rickettsia felis*, Domestic cat, Dog, Whole blood, USA

## Abstract

**Background:**

The main vector and reservoir host of *Rickettsia felis,* an emerging human pathogen causing flea-borne spotted fever, is the cat flea *Ctenocephalides felis*. While cats have not been found to be infected with the organism, significant percentages of dogs from Australia and Africa are infected, indicating that they may be important mammalian reservoirs. The objective of this study was to determine the presence of *R. felis* DNA in the blood of domestic dogs and cats in the USA.

**Methods:**

Three previously validated PCR assays for *R. felis* and DNA sequencing were performed on blood samples obtained from clinically ill domestic cats and dogs from 45 states (2008–2020) in the USA. The blood samples had been submitted for the diagnosis of various tick-borne diseases in dogs and feline infectious peritonitis virus, feline immunodeficiency virus, and *Bartonella* spp. in cats. Phylogenetic comparisons were performed on the *gltA* nucleotide sequences obtained in the study and those reported for *R. felis* and *R. felis*-like organisms.

**Results:**

Low copy numbers of *R. felis* DNA (around 100 copies/ml whole blood) were found in four cats (4/752, 0.53%) and three dogs (3/777, 0.39%). The very low levels of infection in clinically ill animals is consistent with *R. felis* being an unlikely cause of disease in naturally infected dogs and cats. The low copy numbers we found emphasize the requirement for very sensitive PCRs in prevalence studies.

**Conclusions:**

The low prevalence of naturally infected PCR-positive cats is further evidence that cats are unlikely to be important reservoirs of *R. felis*. Similarly, the low prevalence in dogs suggests they are not important reservoirs in the USA. Investigations should continue into the role other mammalian species may be playing in the epidemiology of *R. felis* infections. 
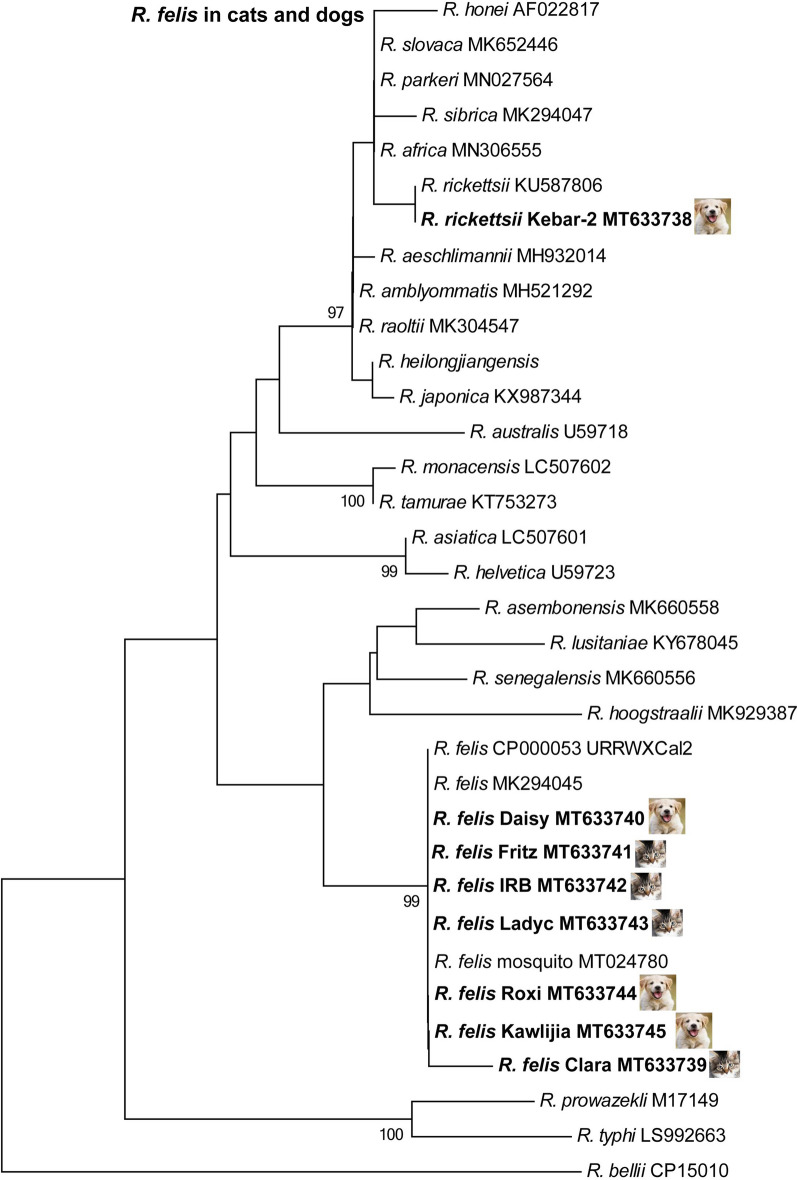

## Introduction

The intracellular bacterium *Rickettsia felis* is the agent of flea-borne spotted fever or cat-flea typhus [1, 2], an emerging zoonotic disease. Since the first report in 1994 of *R. felis* in a person in Texas [3], infections have been described from around the world, with *R. felis* implicated as the causative agent of an important febrile illness in sub-Saharan Africa [4, 5] and flea-borne spotted fever considered a global emerging threat to human health [6].

Various hematophagous arthropods, including a number of flea, tick, and mosquito species, have been found to carry *R. felis* [6–9], but *Ctenocephalides felis felis* (cat flea) is the only confirmed biological reservoir and vector of the infectious agent [7]. High percentages of cat fleas from around the world have been found to be infected with *R. felis—*for example, 30–79% in different U.S. states (Alabama, Maryland, Texas, Oklahoma, and Northern California) [10–12]. Experimental studies have shown that adult *C. felis felis* can become infected by feeding on rickettsemic animals, co-feeding with infected fleas, or as larvae feeding on infected adult feces, eggs, or other larvae [13, 14]. Infections are passed vertically and transstadially in subsequent generations of fleas, and *R. felis* is found in the salivary glands and be transmitted to mammalian hosts during feeding [15].

A number of mammals have been found to be PCR-positive for *R. felis* DNA, mainly dogs [5], opossums [3, 16], raccoons [17], and rodents [5, 16]. Although cats were originally thought to be the mammalian reservoir hosts, experimental infections only revealed brief asymptomatic rickettsemia 2 months post infection, and then only in five of the 16 cats in the study [18]. Although naturally infected seropositive cats have been identified around the world [13], none have been found to be positive for *R. felis* in PCR assays performed on cats from Australia (*n* = 11) [19], China (*n* = 135) [20], Spain (*n* = 212) [21], Thailand (*n* = 585) [22, 23], and the USA (*n* = 121) [10, 12]. Even cats infested with infected fleas are found to be PCR negative [10, 20, 21]. In the USA, Stephenson et al. reported a *Rickettsia* seroprevalence of 3% in people, 42% in dogs, 79% in cats, 33% in gray foxes, and 83% in bobcats, but reverse transcription (RT)-PCR on blood tested consistently negative [12].

Several studies have shown that apparently healthy dogs are not uncommonly PCR positive for *R. felis*, with 3.5% of 200 owned dogs testing positive in Zambia [5], 9% of 100 pound dogs in Australia [1], 2% of 130 indigenous community semi-domesticated dogs in Australia [2], and a dog in the household of a Spanish flea-borne spotted-fever patient [24]. A recent study has shown experimentally infected dogs can be rickettsemic with *R. felis* for at least 3 months and be infectious for fleas, indicating they are a potential mammalian reservoir of the organism [14].

To shed further light on the *R. felis* status of dogs and cats from around the USA, we used PCR assays to analyze DNA from clinically ill cats and dogs from 45 states in the USA.

## Materials and methods

### Whole blood samples

Whole blood samples in EDTA from dogs (*n* = 777) in 45 states of the USA submitted to the Molecular Diagnostics Laboratory at Auburn University College of Veterinary Medicine between 2008 and 2020 for diagnosis of various tick-borne diseases (babesioses, hepatozoonoses, ehrlichioses, and anaplasmoses) were used in the study. We also used whole blood samples in EDTA from domestic cats (*n* = 752) in 43 states submitted for the molecular diagnosis of FIP, FIV, FeLV, and *Bartonella* infections between 2008 and 2020. The samples had been sent to Auburn at ambient temperature and, upon arrival, DNA had been extracted from 800-µL aliquots using the High-Pure PCR Template Preparation Kit (Roche Molecular Biochemicals, Indianapolis, IN, USA) as previously published [25]. The DNA was eluted in 40 µL elution buffer, and the 20-µL volume of each sample remaining after the tick-borne diseases (dogs) or FIP, FeLV, FIV, and *Bartonella* (cats) PCR assays had been performed was preserved at − 80 ºC until the PCR assays to detect *R. felis* were performed in this study.

### Detection of *Rickettsia* DNA by PCR

Three previously validated quantitative PCR assays, mainly a *gltA*-based *Rickettsia* FRET-PCR [26], a nested-PCR targeting the *gltA* of *Rickettsia* [26], and a *R. felis* species-specific *BioB*-based PCR [27], were used to detect *Rickettsia* DNA in the samples. To optimize the sensitivity of our testing and the relatively small amount of sample DNA available to us, we initially screened 10 µL of each DNA sample with the highly sensitive *Rickettsia* FRET-qPCR. The remaining DNA of samples positive in the FRET-qPCR was arbitrarily diluted five-fold with 1× PBS, and 10-µL aliquots were used in the nested and *R. felis* species-specific *BioB*-based PCRs. The *R. felis* DNA from previous studies [26, 28] and nuclease-free water were used as positive and negative controls, respectively. The arbitrary fivefold dilution was also performed on the positive controls.

All PCR reactions were performed on a Roche Light Cycler 480 II thermocycler (Roche Diagnostics GmbH, Mannheim, Germany) as previously described [28]. In brief, 10 µL of the extracted DNA was added to a 10-µL reaction mixture containing 5x PCR FRET buffer, 400 µM dNTP (Roche Diagnostics GmbH), 0.34 units of Platinum *Taq* DNA Polymerase (Invitrogen, Carlsbad, CA, USA), 1 µM of each forward and reverse primer (Integrated DNA Technologies, Coralville, IA, USA), and a final volume of molecular grade nuclease-free water.

**Fig. 1:**
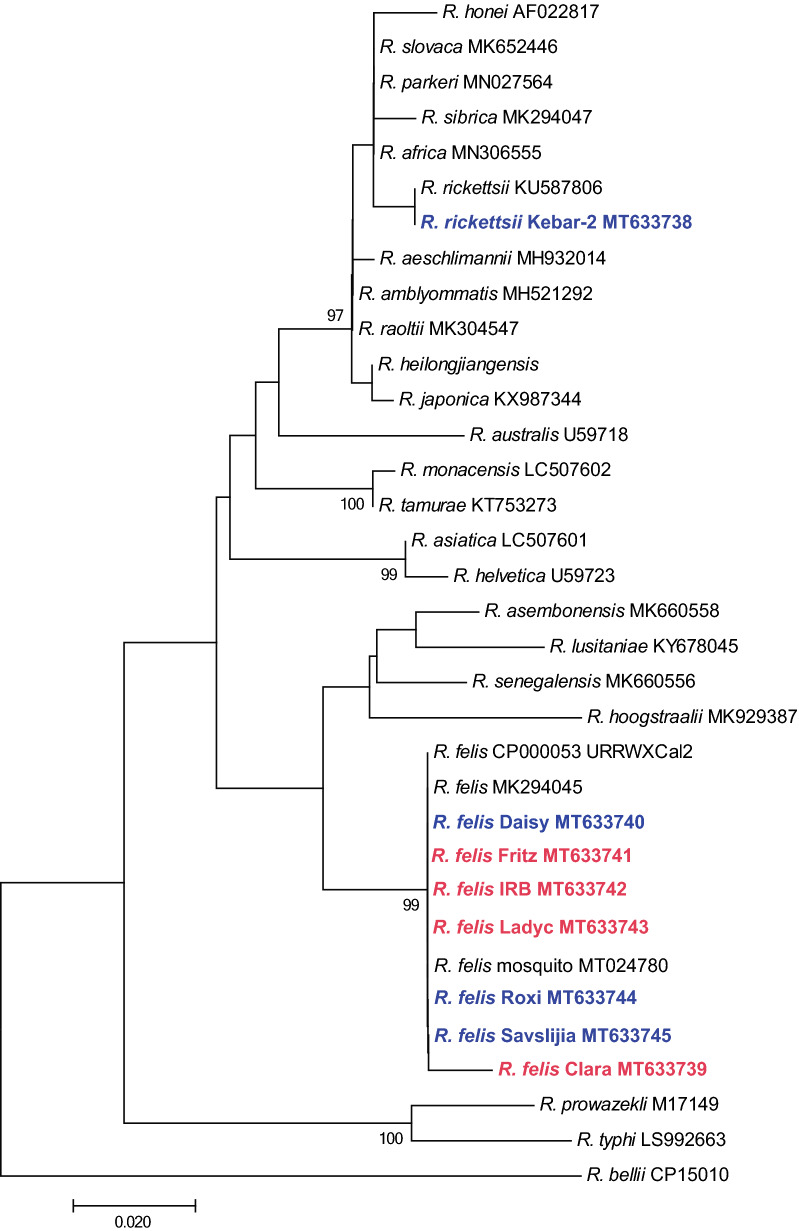
Phylogenetic tree using a bootstrap analysis for the *Rickettsia felis* found in mosquitoes from the USA. The names of *Rickettsia* species and their GenBank Accession numbers are provided. The sequences of *R. felis* identified in this study (in bold; red for cats and blue for dogs) were 99.0–100% identical to the recognized *R. felis* strains, 94.0% (*R. asembonensis*) and 95.7% (*R. senegalensis*) identical to *R. felis*-like organisms, and 84.0–94.7% identical to other *Rickettsia* spp.

The products of *Rickettsia*-positive PCRs were sent to ELIM Biopharmaceuticals (Hayward, CA, USA) for Sanger DNA sequencing using upstream and downstream primers. The nucleotide sequences were submitted to the National Center for Biotechnology Information (NCBI) to obtain GenBank Accession numbers, and a phylogenetic tree was generated to compare the nucleotide sequences of *Rickettsia* identified in this study with those of *R. felis*-like organisms [29] and other *Rickettsia* species (Fig. 1). The nucleotide sequences of the *gltA* PCR products were concatenated and aligned using CLUSTALW, and the phylogenetic inferences were obtained from a maximum likelihood analysis.

## Results

The *Rickettsia* FRET-qPCR identified *Rickettsia* DNA in nine cats (1.2%) and eight dogs (1.0%). Sequencing confirmed the presence of *Rickettsia*, but the relatively short amplicon (170 bp) produced by the FRET-qPCR did not enable the reliable differentiation of the species present. Of the 17 FRET-qPCR-positive samples, eight were positive in the nested PCR assays performed with the fivefold diluted DNA (Table 1). Four of these diluted DNA samples were also positive in the *R. felis*-specific *BioB*-based PCR assay.

**Table 1:** Identification of *Rickettsia* spp. in whole blood of cats and dogs by PCR assay

Sample ID	Host	State	Year	Background	FRET PCR	Nested PCR	*BioB* PCR
Ladyc	Cat	Manhattan, KS	May 2013	5-year; female, spayed; for *Bartonella*-negative	*R. felis*	*R. felis*	*R. felis*
IRBE32218089	Cat	Granada Hills, CA	July 2014	5-year; male castrated; Siamese; for FIP-negative	*R. felis*	*R. felis*	*R. felis*
Clara	Cat	Brooklyn, NY	Nov 2015	American domestic shorthair; 8-month; female; for FIP-negative	*R. felis*	*R. felis*	*R. felis*
Fritz	Cat	Spring, TX	2017	7-month; American domestic shorthair; male, castrated; for FIP-negative	*R. felis*	*R. felis*	*R. felis*
Rebar	Dog	Montgomery, AL	June 2013	Male; for *Hepatozoon*-negative	*R. rickettsii*	*R. rickettsii*	Failed
Daisy	Dog	Killeen, TX	Nov 2017	Two-month shifting leg lameness. Jack Russell Terrier Mix; female, spayed; 6Y. For *Hepatozoon*-negative	*R. felis*	*R. felis*	Failed
Roxie	Dog	Jefferson, GA	Dec 2014	Bulldog; female; 6-year; chronic weight loss with muscle/joint pain; for *Hepatozoon*-negative	*R. felis*	*R. felis*	Failed
Kawlija	Dog	Rome, GA	Dec 2015	Jack Russell Terrier; 11-year; male; for *Hepatozoon*-negative; 6-week history of vomiting/diarrhea; intermittent pain	*R. felis*	*R. felis*	Failed

FIP, Feline infectious peritonitis; FRET, fluorescence resonance energy transfer,* R.*, *Rickettsia*

Sequencing of the amplicons of the positive nested PCR assays amplifying the *gltA* revealed that four cats (4/752, 0.53%; standard deviation [SD] 0.073) and three dogs (3/777, 0.39%; SD 0.062) were positive for *R. felis* and one dog was positive for *R. rickettsii* (Table 1). Six (from 3 dogs and 3 cats) of the seven 300-bp *gltA* nucleotide sequences were identical to the recognized *R. felis*-type strain (CP00053 URRWXCaL2) (Fig. 1) with one, from a cat, having only a single nucleotide difference to the type strain. The sequences of *R. felis* identified in this study had a lower similarity to the *R. felis*-like organisms (94.0% to *R. Asembonensis*; 95.7% to *R. senegalensis*), and only 84.0–94.7% similarity to other *Rickettsia* spp. (Fig. 1).

The *R. felis*-specific *BioB*-based PCR assay was positive for all four of the *R. felis*-positive cat samples but was negative for the canine samples (Table 1).

The copy number in the FRET-qPCR assay for the *R. rickettsii*-positive dog sample was very high (5 × 10^6^ copies/mL whole blood) compared with those for the *R. felis*-positive cats and dogs (about 100 copies/mL whole blood).

The four *R. felis*-positive cats were each from different states: Kansas (sample was submitted in May 2013), California (July 2014), New York (July 2015), and Texas (November 2017). All of the samples were negative by PCR for feline coronavirus and *Bartonella* spp. for which they had originally been submitted for testing (Table 1).

Three *R. felis*-positive dogs were from Texas (November 2017) and Georgia (December 2014; December 2015), while the *R. rickettsii-*positive dog was from Alabama (June 2013). All of the dog samples had originally been submitted for PCR testing for *Hepatozoon* spp., and all were negative. Of note: all three *R. felis*-positive dogs were suffering from muscle and joint pain (Table 1).

## Discussion

*Rickettsia felis* occurs widely around the world, and recent findings that the organism might also be transmitted by mosquitoes [28] have raised the possibility that human flea-borne spotted fever might be the next mosquito-borne pandemic [30]. Expanding our understanding of the mammalian hosts of *R. felis* will be important in developing strategies to prevent and control potential outbreaks.

This study adds to existing data that *R. felis* occurs widely in the USA. To the best of our knowledge, this is the first description of PCR-positive naturally infected domestic cats. However, only very few cats (4/752) tested positive, which casts even further doubt on a possible role cats might play as reservoirs of *R. felis*.

Although dogs have commonly been found to be PCR-positive for *R. felis* in Australia [1, 2], we found only low numbers of positive dogs in our study. Previously, we also found only a low percentage (0.8%) of dogs (*n* = 11,059) from China to be PCR positive despite 47% (128/271) being seropositive [20], about the same proportion as the 51% in Australia where 2–9% of dogs were PCR positive [31]. Similarly, in a study in northern California, Stephenson et al. [12] did not identify *R. felis* DNA in the blood of dogs (0/163), although 48% were seropositive against *R. rickettsii* and 39% of *C. felis* (*n* = 152) collected from the dogs were PCR positive. It is of note, however, that it has recently been shown that dogs are reservoirs of *R. felis*, having asymptomatic rickettsemia for up to 100 days after experimental infection and being a source of infection for naïve fleas [14]. The growing body of available data indicates that the importance of dogs in the epidemiology of *R. felis* might vary from area to area. To more precisely determine the role dogs play in the epidemiology of *R. felis*, further studies are needed which consider factors such as dog breed, level of flea infestation, nutritional status, and presence of other vector-borne diseases.

Although, to the best of our knowledge, this is the first description of naturally infected domestic cats that were PCR positive for *R. felis* , actually only very few animals (4/752) were positive, which casts even further doubt on a possible role cats might play as reservoirs of *R. felis*. While the lower level of infections in cats might be because cats are less susceptible to infections, it might also be because cats are only rickettsemic for very short periods before mounting immune responses that clear infections [18]; in contrast, in dogs, circulating *R. felis* may persist for over 3 months [14], which would increase the likelihood of detection in random PCR surveys.

This study is the first to report copy numbers of *R. felis* DNA in dogs and cats. It has previously been suggested that negative results in *R. felis* surveys might have resulted from (1) standard PCR assays having low sensitivity and (2) there being only low circulating levels of the microorganism [10]. The latter possibility appears to be true, as we found only low copy numbers (around 100 copies/mL whole blood) in positive cats and dogs. The FRET-qPCR we developed proved to be very sensitive, detecting one gene copy per 20 µL of reaction system [20], and the use of this FRET-qPCR, or at least of equally sensitive PCR assays, appears to be indicated in future studies of mammalian reservoirs of *R. felis*. Although we did not study how copy numbers varied over time, the low copy numbers recorded make it appear likely that dogs and cats can only be efficient reservoirs of infection if there are large populations of fleas and if those fleas are very susceptible to infection.

Our study population consisted of cats and dogs that were clinically ill, with signs suggestive of tick-borne diseases in the dogs and of *Bartonella*, FIV, or FeLV infections in the cats. The fact that only relatively few of these clinically ill cats and dogs were PCR positive for *R. felis* is consistent with the existing data that infections are typically asymptomatic [1, 2, 5, 18, 31, 32] and that *R. felis* is an unlikely cause of fever in cats [33].

## Conclusion

In conclusion, this study adds to the body of available data showing that cats are not likely to be important reservoirs of *R. felis* in the USA. Although dogs have been shown to be reservoirs of *R. felis*, the available evidence indicates this might not be the case in the USA. Further studies are needed to identify the factors, such as breed, concurrent disease status, and flea burdens, that would enable dogs to play an important role in the epidemiology of *R. felis* in the USA. Control of human infections must still be directed at the control of fleas on domestic animals as the available evidence indicates these are the major reservoirs and vectors transmitting *R. felis.* Investigations should continue into the role that other mammalian species may be playing in the epidemiology of *R. felis* infections.

## Data Availability

Data supporting the conclusions of this article are included within the article.

## Abbreviations

FeLV: Feline leukemia virus
FIP: Feline infectious peritonitis
FIV: Feline immunodeficiency virus
FRET: Fluorescence resonance energy transfer
